# Surface Segregation of Cyclic Chains in Binary Melts of Thin Polymer Films: The Influence of Constituent Concentration

**DOI:** 10.3390/polym10030324

**Published:** 2018-03-15

**Authors:** Francis M. Gaitho, Mesfin Tsige, Genene T. Mola, Giuseppe Pellicane

**Affiliations:** 1 School of Chemistry & Physics, University of KwaZulu-Natal, Pietermaritzburg, Private Bag X01, Scottsville 3209, South Africa; fgaitho@mmust.ac.ke (F.M.G.); mola@ukzn.ac.za (G.T.M.); 2 Department of Polymer Science, University of Akron, Akron, OH 44325, USA; mtsige@uakron.edu; 3 National Institute of Theoretical Physics (NITheP) KZN Node, Pietermaritzburg, Private Bag X01, Scottsville 3209, South Africa

**Keywords:** polymer melt, concentration, surface behavior, interface

## Abstract

We carry out extensive molecular dynamics simulations of thin films of bead-spring models of binary mixtures composed of cyclic and linear polymer chains. We study the equilibrium behavior of the polymer chains for two very different chain lengths, which resemble short (10-mers) and long (100-mers) chains, at different concentrations of the binary mixture. We clearly show how the concentration variable affects the enrichment of either of the two polymer species at the interface, and also how the chain length influences this process.

## 1. Introduction

The dynamics and phase separation of binary polymer blends is in general dependent on the concentration of one of the two molecular species in the medium. Currently, many commercial polymer-based optoelectronic devices are fabricated using polymer blends [1,2], and their technological applications range from plastics industry [3,4,5,6], to organic photovoltaics [7,8] (where fullerene-derived [9] molecules are also used). As the surface of these materials is approached, and the polymer blend is exposed to air/vacuum, or to a porous substrate, any understanding of the role played by concentration becomes even more complicated by the presence of an inhomogeneous environment. Consequently, gaining microscopic insight into how the change in concentration affects interfacial properties is necessary to predict mechanical and electrical behavior of polymeric materials, as well as to control many processes of industrial interest, including chemisorption, oxidation, and corrosion [10,11].

Several computational [12,13,14,15,16,17,18,19,20] and experimental [21,22,23,24,25] investigations of interfacial properties of thin films of binary polymer blends have been reported in recent literature. In general, variations in the local concentration of the binary blend in proximity to the interface with respect to the concentration in the *bulk* region of the material, is explained in terms of several features. Some of them include different chemical properties and molecular weights [26] of the two molecular species, and the different enthalpic, attractive forces they experience towards the substrate. However, when polymers with different topologies are mixed to form a blend, and the monomer chemistry of the two polymers is similar, this picture cannot be used anymore. Recently, numerical work has been published to provide some insight into the microscopic mechanisms governing topologically-driven preferential adsorption of one of the two polymers at the interface [27,28,29]. Specifically, one system that has been subject of intense study is a blend of linear and cyclic polymers, where the strategy of mixing up a closed-loop and an open-loop polymer architecture is probed as a microscopic tool for modifying the interfacial properties [14,30]. The interest in computational models of linear-cyclic polymer blends is because these blends have been engineered recently thanks to new advances in synthetic chemistry [30,31,32]. However, numerical and experimental studies of this system were conducted mainly in the limit where cyclic-chains are less concentrated than linear ones, while the effect of changing the concentration over a more extended range is still unexplored.

The main aim of this paper is to fill the gap of knowledge regarding the effect of concentration on interfacial properties of cyclic-linear polymer mixtures exposed to vacuum, by performing extensive molecular dynamics (MD) simulations. We also change the chain lengths of the polymer species, which, for these systems, was shown to play an important role [30,33], by considering the two limiting cases of short and long chains (10-mers and 100-mers, respectively).

The paper is organized as follows: we introduce the model and the computer simulation approach in Section 2, followed by a discussion of our results and findings in Section 3. Finally, the summary and conclusion of the investigation are presented in Section 4.

## 2. Model and Simulation Details

### 2.1. The Bead-Spring Model

To study the behavior of cyclic and linear polymers at the polymer/vacuum interface, we used the Kremer–Grest bead-spring model [34]. The choice of this model was motivated by its flexibility to be adapted for studying a large class of polymeric materials. In this model, polymers are considered as chains of sequential monomers with equal mass (m), each connecting with the previous one to form either cyclic (closed loop) or linear (open loop) chains. The interaction potential between non-bonded monomers is described by the standard 12-6 Lennard–Jones (LJ) potential [34,35], that was truncated and shifted at $r_{c}=2.5\sigma$:

(1) $$E_{LJ}\left(r\right)=\left\{\begin{array}{cc} 4\epsilon \left[{\left(\frac{\sigma }{r}\right)}^{12}-{\left(\frac{\sigma }{r}\right)}^{6}\right]+\epsilon_{LJ}, & r\leq r_{c}\\ 0 & r>r_{c} \end{array}\right.$$

where *r* is the distance between monomers, ϵ is the potential energy at the minimum, σ is the Lennard-Jones parameter, and $\epsilon_{LJ}$ is an energy constant added so that the potential is continuous at $r=r_{c}$. Bonded monomers interact with the LJ potential reported in Equation (1), plus a pair potential energy function known as the finitely extensible nonlinear elastic (FENE) potential [16,36]:

(2) $$E_{f}\left(r\right)=\left\{\begin{array}{ll} -\frac{1}{2}Kr_{0}^{2}\ln\left[1-{\left(\frac{r}{r_{0}}\right)}^{2}\right], & r\leq r_{0}\\ \infty & r>r_{0} \end{array}\right.$$

where $r_{0}$ = 1.5σ and *K* = $30\epsilon /\sigma^{2}$. No angle potential among three consecutive beads was applied. Finally, Brownian and frictional forces are acting on polymer beads [34,35].

### 2.2. Simulation Details

The initial configurations were generated by randomly placing cyclic and linear polymers of equal chain lengths in a parallepiped simulation box. In the present study, we study cyclic and linear polymers consisting of 10 and 100 beads per chain. The concentration $C_{0}=100\times N_{c}^{t}/\left(N_{c}^{t}+N_{l}^{t}\right)$ of cyclic chains in the blend is varied as $C_{0}$ = 10%, 30%, 50%, 70% and 90%, where $N_{c}^{t}$ and $N_{l}^{t}$ are the total number of cyclic and linear polymer beads in the simulation box, respectively. The systems studied here have a total number of beads $N^{t}$ = $10^{5}$ for 10-mer chains, and $N^{t}$ = 2 ×$10^{5}$ for 100-mer chains.

All simulations were performed using the LAMMPS simulation package [37]. The equations of motion were integrated using a velocity-Verlet algorithm with a time step of Δt=0.005τ, where $\tau ={\left(m\sigma^{2}/\epsilon \right)}^{1/2}$. The initial configurations were first equilibrated for more than 50,000τ in the NPT ensemble at P = 0 and T = $\epsilon /k_{B}$, using a Langevin thermostat and a Berendsen barostat with periodic boundary conditions in all three directions. Periodicity along the *z*-direction was then removed and the box elongated in that direction to generate thin films exposed to nearly the same volume of empty space, both at the bottom and the top of the simulation box, respectively. The new simulation box dimensions after equilibration were: $L_{x}=L_{y}=62.4\sigma$ for *N* = 10 and $L_{x}=L_{y}=84.0\sigma$ for *N* = 100, while $L_{z}=180.0\sigma$ for both cases.

Simulations were then performed in the NVT ensemble using the Langevin thermostat [38,39] for $10^{6}\tau$ and quantities of interest were averaged over the last 250,000τ. We verified that the density in the middle of the films after equilibration closely matched the *bulk* one at the end of the NPT equilibration runs. The *bulk* density of the system within the NPT simulations is defined as the ratio between the total number of particles in the simulation box, divided by the volume of the box at the end of the equilibration run. In the simulations where the blend is exposed to empty space along the *z*-axis, and two empty space-blend interfaces are formed at low and high *z*-values, the *bulk* properties of the system are operatively estimated in the slab of size σ along the *z*-direction, that is located at the middle distance between the two interfaces. All the physical quantities reported in the next section were calculated inside slices (bins) of size σ along the *z*-direction of the simulation box. The partitioning of the simulation box in slices of size σ started at the value of the *z* coordinate of the polymer bead inside the simulation box, that is more exposed to empty space, so that two different grids were used for the two interfaces. Values of structural and thermodynamic quantities were averaged in each slice starting from each polymer/vacuum interface, and moving towards the center of the simulation box. Then, in the figures reported in the next section, unless otherwise specified, the origin of *z*-axis coincides with the *z*-position of polymer bead with the lowest (or highest) *z* value contained inside the simulation box.

## 3. Results and Discussions

### 3.1. Density and Radius of Gyration

Our approach in characterizing the packing and conformation of the polymer chains in the blend in proximity to the polymer/vacuum interface follows our earlier work [33], in which only the case at equimolar concentration was considered. In this section, we demonstrate that the surface morphology of the films can be characterized by two properties, namely, local density and radius of gyration of the chains, which we use to investigate the arrangement and shape of the polymer chains at the different concentrations. For some selected cases, we run MD simulations well beyond 250,000τ to safely capture the conformation and structuring of polymer chains at the interface under equilibrium conditions.

#### 3.1.1. Density

**Figure 1:**
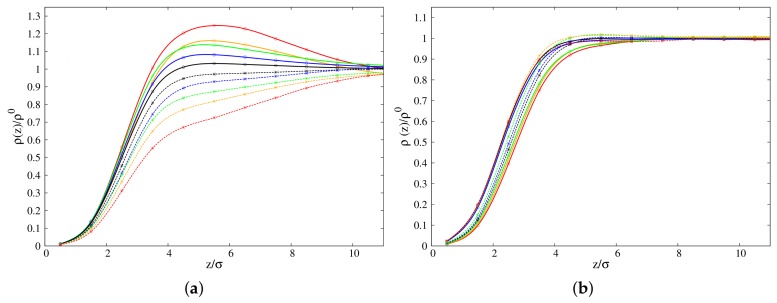
Density profiles of polymer films of (**a**) 100-mers and (**b**) 10-mers at concentrations $C_{0}$ = 10% (Red lines), 30% (Orange lines), 50% (Green lines), 70% (Blue lines) and 90% (Black lines). Cyclic chains: solid lines; linear chains: dashed lines.

We define the local density ρ(z) as $\rho_{l}$, for linear species, and $\rho_{c}$, for cyclic species, by the expression

(3) $$\rho_{\left(l,c\right)}=\frac{N_{\left(l,c\right)}}{V}$$

where $N_{\left(l,c\right)}$ is the number of beads of linear/cyclic polymer species contained in a slab of thickness σ along the z-direction, and *V* is the volume of that slab. We considered variations of the density ρ(z) at different concentrations of cyclic-linear polymer blends for 10-mers and 100-mers, by scaling the local density with respect to the *bulk* value $\rho^{0}$. Values of scaled densities, ρ(z)/$\rho^{0}$, as function of distance *z* from the polymer/vacuum interface, are presented in Figure 1a,b. Inspection of Figure 1a (100-mers) shows increase of the scaled densities at the interface for linear chains, and decrease of the same quantity for cyclic chains, as the concentration of cyclic chains is increased. Alternatively, the scaled density of both polymer species at the interface increases, as the concentration of each component is decreased.

The higher values for the scaled density of cyclic chains nearby the interface are consistent with cyclic chains being more enhanced than linear chains at the surface, in agreement with what was observed for some selected concentrations previously [33]. While the present results for the long chain case confirm that cyclic-chains are preferentially absorbed at the interface, the results reported in Figure 1a provide us with a new and interesting insight about the way the concentration of the different components affects surface behavior. In fact, at the lower cyclic concentration, the gap between the cyclic and linear density profiles nearby the interface is maximum, i.e., cyclic adsorption is more enhanced. Increasing the cyclic concentration makes the gap smaller, but still detectable even at the highest considered concentration $C_{0}=0.9$. These numerical results provide a rationale for the evidence that experiments are conducted mainly in the regime of dilute cyclic concentration, where according to our results it should be easier to detect the preferential adsorption of cyclic chains at the interface. Another interesting feature is that closer to the interface, at all concentrations, the scaled density of cyclic chains is more enhanced than that of linear chains over a range of distances of *z*≈ 9–10σ from the interface, beyond which the density of both chains converges to the corresponding *bulk* value in the blend. In the case of small chains (10-mers), the results reported in Figure 1b show an opposite behavior as the one observed for the long chain case at the interface: decrease of the scaled densities at the interface for linear chains, and increase of the same quantity for cyclic chains, as the concentration of cyclic chains is increased. Alternatively, a general increase (with some minor deviations) in the scaled density is observed for both species when the concentration of each component is in turn increased. However, only the gap between the cyclic and linear density profiles in the regime of dilute cyclic concentration up to the equimolar case is still evident, and it clearly shows preferential linear adsorption at the interface, which is in agreement with what was reported in previous papers [14,33]. In the regime of more concentrated cyclic polymers, a slight preferential adsorption of cyclic chains emerges similarly as in the previously considered case of long-chains (see Figure 1a). Another feature evident in Figure 1b is that the change in density with concentration is not as dramatic as was shown for the 100-mers case in Figure 1a, and there is no net separation between the cyclic and linear density profiles nearby the interface.

A noteworthy feature for cyclic chains of 100-mers is a maximum (peak) at z≈ 4–6σ, in agreement with a similar evidence as reported in our recent study on symmetric linear-cyclic blends [33]. At this peculiar distance from the interface, attraction between cyclic molecules generates geometrical constraints (we will discuss this aspect later), thus preventing close packing of cyclic chains in the *bulk* of the blend. In the recent literature [40,41], preferential adsorption of polymer components at the interface (in the absence of preferential interaction energies with the medium at the interface) was shown to arise from competition between configurational energy and entropic packing effects. Here, we show that concentration can also be another important parameter affecting enhancement/depletion of polymer species of a blend at the interface. For comparison, *bulk* density values of both types of chains are presented in Table 1.

#### 3.1.2. Radius of Gyration

In this section, we focus on a well-known structural property of polymer chains. We use the average radius of gyration, $\bar{R_{g}}$, to study the conformation of polymer chains at the polymer/vacuum interface:

(4) $$\bar{R_{g}}=\sqrt{\langle \frac{1}{N}\sum_{i=1}^{N}{\left(\vec{r_{i}}\left(t\right)-{\vec{r}}_{com}\left(t\right)\right)}^{2}\rangle }$$

where the bar over the symbol $R_{g}$ indicate an average over time, *N* is the total number of beads per polymer, $r_{com}\left(t\right)$ is the position of center-of-mass of the whole chain and $r_{i}\left(t\right)$ is the position vector of each bead in a chain at time t. Finally, the brackets <…> indicate an average over all the chains contained inside the simulation box.

**Figure 2:**
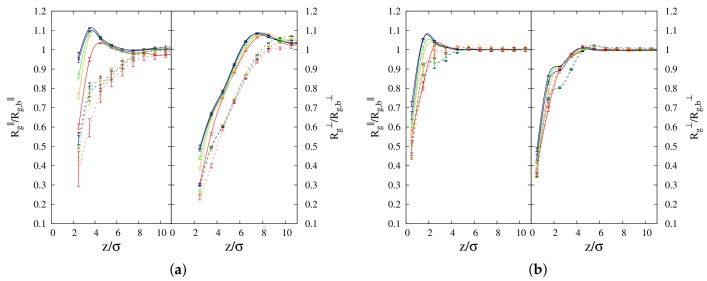
Profiles for radius of gyration $R_{g}$ as a function of distance from interface *z*. Full lines: cyclic chain profiles. Dashed lines: linear chain profiles. Left panels: Parallel components $R_{g}^{∥}$, for (**a**) 100-mers and (**b**) 10-mers, at concentrations $C_{0}$ = 10% (Red lines), 30% (Orange lines), 50% (Green lines), 70% (Blue lines) and 90% (Black lines). Right panels: Transverse component $R_{g}^{⊥}$—for the same order of presentation as in the left panels. $R_{g}^{∥,⊥}$ are scaled by their *bulk* values $R_{g,b}^{∥,⊥}$.

**Figure 3:**
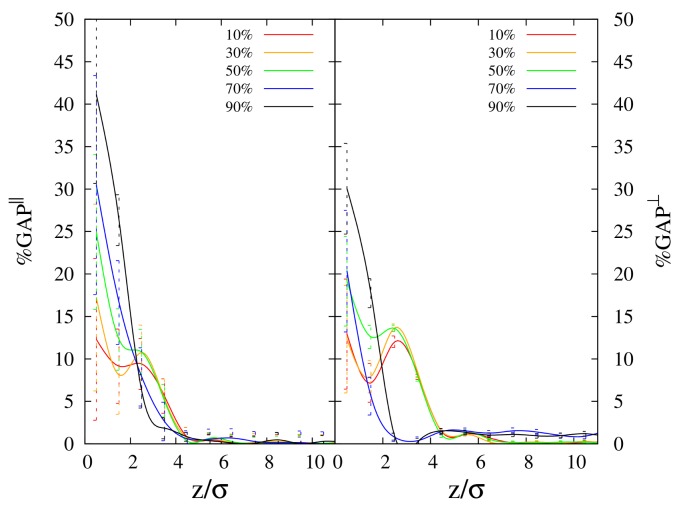
Profiles for average gap of radius of gyration between cyclic and linear polymers as a function of distance from the interface *z*, for 10-mers. $GAP^{∥}$- left panels; $GAP^{⊥}$- right panels.

The parallel ($R_{g}^{∥}$) and perpendicular ($R_{g}^{⊥}$) components of $R_{g}$ are defined as

(5) $$\bar{R_{g}^{∥}}=\sqrt{\langle \frac{1}{N}\sum_{i=1}^{N}\left[{\left(x_{i}\left(t\right)-x_{com}\left(t\right)\right)}^{2}+{\left(y_{i}\left(t\right)-y_{com}\left(t\right)\right)}^{2}\right]\rangle }$$

(6) $$\bar{R_{g}^{⊥}}=\sqrt{\langle \frac{1}{N}\sum_{i=1}^{N}{\left(z_{i}\left(t\right)-z_{com}\left(t\right)\right)}^{2}\rangle }$$

and they are reported as a function of distance from the interface in Figure 2a,b, for 100-mers and 10-mers, respectively. In this case, the procedure described at the end of the subsection “Simulation details” is applied by localizing the position of the center of mass of each polymer inside a slice of size σ. By looking at $R_{g}^{∥}$ (Figure 2a) close to the polymer/vacuum interface, we observe enhanced values for cyclic chains in comparison to linear chains. Regardless of the chain length (see left panels of Figure 2a,b), $R_{g}^{∥}$ of both cyclic and linear polymers increases with increase in cyclic chain concentration. While the curves for cyclic and linear chains are clearly separated for long chains (see Figure 2a), in the case of short chains, they appear as overlapping with each other. Then, to better understand the trend with varying cyclic concentration, for the case of 10-mers, we monitored the average gap between $R_{g}^{∥}$ of the two polymer species at the interface, as a function of cyclic concentration. In Figure 3, we report the quantity $\%GAP=200\cdot \left|\frac{R_{g}^{c}/R_{g,b}^{c}-R_{g}^{l}/R_{g,b}^{l}}{R_{g}^{c}/R_{g,b}^{c}+R_{g}^{l}/R_{g,b}^{l}}\right|$ for the parallel and transverse components of the radius of gyration, where $R_{g}^{c}$ and $R_{g}^{l}$ are the corresponding quantities of cyclic and linear chains, respectively. In the definition of %GAP, we scaled the absolute gap value (numerator of the fraction) of $R_{g}$ between the two species, by the corresponding average radius of gyration of the two polymer species (that is the reason of the factor 200 appearing in its definition, i.e., 100×2). That allowed us to detect that, for the smaller chain length (see left panel of Figure 3), the average gap between $R_{g}^{∥}$ of the two polymer species at the interface tends to increase as we increase the cyclic concentration. This effect is compatible with what we observed before for the reduced density (see Figure 1b), where we showed that linear chains get less adsorbed at the interface when we increase the cyclic concentration. In fact, the conformational entropy of cyclic chains gets comparatively higher than the one of linear chains as we increase cyclic concentration, since the relative difference in the radii of gyration of the two polymer species increases as well (see Figure 3). In the case of long chains (100-mers), the linear chains near the interface are clearly observed to get more folded than their cyclic counterparts, along the longitudinal direction to the interface (see Figure 2a). This evidence confirms that the higher loss of conformational entropy of linear polymers for long chains is a factor preventing their enrichment at the interface [14] over the whole concentration range of the mixture. A peak in $R_{g}^{∥}$ of cyclic chains is also observed at all concentrations, which moves slightly closer to the interface as the concentration of cyclic chains is enhanced, starting from z≈ 3–4σ from the interface for 100-mers, and slightly earlier at z≈ 2–3σ for 10-mers. Not only is the position of the peak sensitive to the chain length, but also its height is enhanced when the cyclic concentration increases, for both chain lengths, up to a maximum of $R_{g}^{∥}$ ≈1.15 for 100-mers, and $R_{g}^{∥}$ ≈1.09 for 10-mers. As already reported before [14,33], long cyclic chains assume a more elongated shape near the interface, but now our results show that this feature amplifies with the increase of the concentration of cyclic chains (peak enhancement of $R_{g}^{∥}$). That is compatible with the likely physical origin, i.e., the topological excluded volume interaction (repulsion) of blobs in a ring of pure cyclic chains [16]. This effect is also a subject of interest in polymer processing using chain topology controlled hyperbranched polyethylene [42]. In the case of $R_{g}^{⊥}$, (right panels of Figure 2a,b), we note that the profiles reach the *bulk* value within approximately $2R_{g,b}$ (see Table 2). The values of the radii of gyration reported in Table 2 also indicate that there is no evident dependency of the *bulk* values $R_{g,b}$ on the concentration of the mixture.

Similar to the case of the parallel components $R_{g}^{∥}$, we observe that the values of $R_{g}^{⊥}$ of cyclic chains are clearly larger than the ones of linear chains for 100-mers, but the same feature does not emerge clearly for 10-mers. However, when we consider the quantity %GAP (right panels of Figure 3) in the case of short chains (10-mers), we can still observe a general trend of enhancement of the radius of gyration of cyclic chains in comparison to linear chains, as we increase the cyclic chain concentration. Once again, at the interface, the comparatively higher conformational entropy of cyclic chains as the cyclic concentration is increased, is compatible with the depletion of linear chains in comparison to cyclic chains, as shown in Figure 1b.

The transverse components of $R_{g}$ for short chains are only slightly dependent on the concentration (see right panel of Figure 2b), and the variation of transverse and parallel components near the interface are of a similar magnitude for both the two polymer species. On the contrary, long cyclic chains (100-mers) exhibit a clear tendency to be elongated parallel to the interface as the concentration of cyclic chains increases from $C_{0}=10\%$ to 90% ( compare the two panels in Figure 2a). In summary, in the case of longer chains, our results show that upon increasing the concentration of cyclic chains, conformational entropic effects play a more important role in determining surface behavior.

### 3.2. Instantaneous Surface Profile

**Figure 4:**
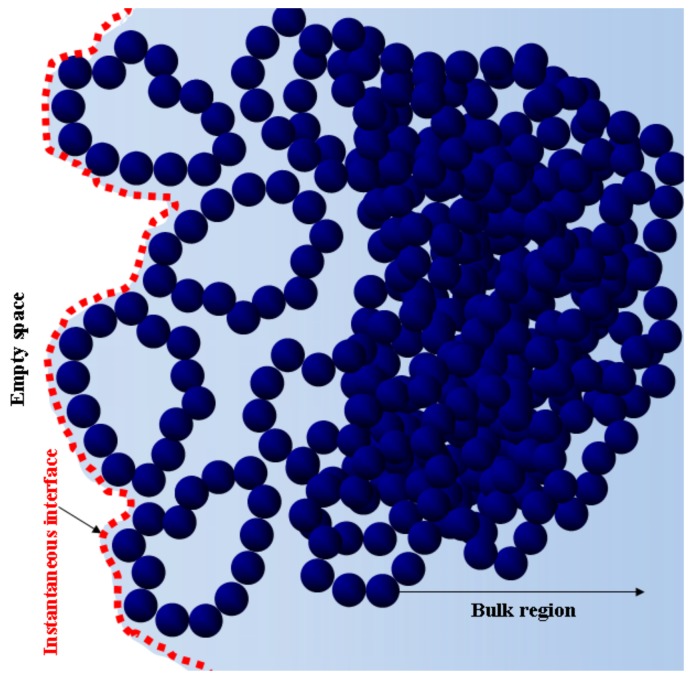
Schematic representation of instantaneous interface generated by cyclic polymers.

It is very challenging to unequivocally define the polymer/vacuum interface due to the dynamical nature of the interfacial configurations arising from surface capillary waves. Consequently, the identity of the species and the position of the vacuum/polymer interface change both spatially and as a function of time. To identify the preferred species at the interface, it is important to properly take into account the fluctuating nature of the interface separating the blend from empty space. We adopted a simple strategy to build a time-averaged number histogram for the particles being targeted, called the instantaneous interface procedure, that has already been discussed in our previous paper [33]. In Figure 4, we show a graphical sketch of the typical spatial arrangement of a polymer species, which helps to understand the issue arising from the roughness of the interface.

**Figure 5:**
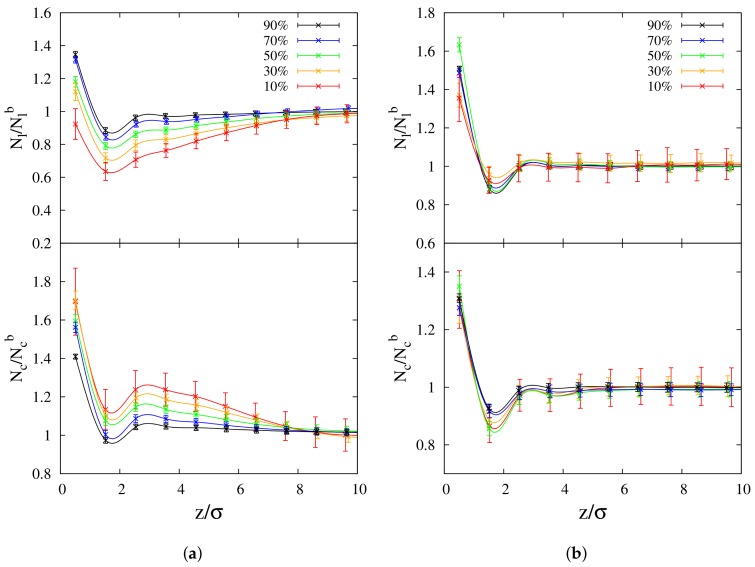
Average polymer numbers for (**a**) 100-mers and (**b**) 10-mers scaled by total number of polymers in the slab of thickness σ in the *bulk*, as a function of distance from the instantaneous interface. $N_{l}$ represents number of beads of linear chains and $N_{c}$ that of cyclic chains.

Using the instantaneous interface procedure, we first characterize the packing of polymer chains near the interface by computing the relative number of linear and cyclic polymers in every slab with respect to the *bulk* value, as

(7) $$P{}_{c,l}=\frac{N_{c,l}}{N_{c,l}^{b}}$$

where $N{}_{c,l}$ is the total number of either cyclic or linear beads within the slab of thickness σ starting from the instantaneous interface, and $N_{c,l}^{b}$ represents the number of beads in a generic slab of the same thickness in the *bulk* of polymer blend. Variations of $P{}_{c,l}$ with distance from the instantaneous interface for 100-mers and 10-mers are reported in Figure 5a,b, at different concentrations.

For 100-mers, a substantial number of cyclic polymers is observed closer to the interface. The values of $P{}_{l}$ at the interface increase from ≈0.9 to 1.3, while $P{}_{c}$ decreases from ≈1.7 to 1.3 as $C_{0}$ increases from 10% to 90% (Figure 5a). This confirms the earlier findings [14] of cyclic chain enhancement at the interface for the longer chain length. However, the novel result emerging here is that the enhancement of cyclic polymers at the interface decreases when $C_{0}$ is increased towards the limit of dense cyclic solutions at $C_{0}=0.9$. In fact, the gap between $P{}_{c}$ and $P{}_{l}$ shrinks when the cyclic concentration increases (see Figure 5a).

Values of $P{}_{l,c}$ for 10-mers do not show a clear trend as a function of $C_{0}$ (see Figure 5b), however we observe that on the average the values of $P{}_{l}$ at the interface are ≈1.4, while values of $P{}_{c}$ are way lower, i.e., ≈1.3. Thus, even the information coming from a refined approach considering the roughness of interface allows us to safely conclude that linear chains are systematically enriched for small chain lengths. In general, the profiles reported in Figure 5a,b show that for long chains, and in the limit of low cyclic concentration, the enhancement of cyclic chains at the instantaneous interface is much larger than the one of linear polymers for short chains. In fact, that is especially evident if we look at the bottom panel of Figure 5a, where enhancement of cyclic chains takes place over an extended range of distances from the instantaneous interface. On the other hand, by looking at the top panel of Figure 5b, we observe that linear chains are enhanced only very close to the instantaneous interface.

The spatially-extended enhancement of cyclic chains from the instantaneous interface could be explained by the observed topological excluded volume repulsion of blobs in a ring [16,43] that makes the cyclic chains more elongated along the interface [33]. Lee et al. [16] described a mechanism where the confined rings crowd out the neighbouring molecules and become more compact by squeezing along the interface. Since this effect is expected to increase with the chain length (see Figure 14 of Ref. [16]), it would allow cyclic polymers to achieve a denser packing structure than linear ones in the region close to the interface. We also note that, very close to the interface, for all the considered cases in Figure 5a,b, there is a minimum in the curves at distances between σ and 2σ. However, that minimum is not genuinely associated with a depletion region for cyclic long chains, since in that case the minimum falls at values above 1 (see bottom panel of Figure 5a).

The location of chain-end beads of linear chains in the blend is also of interest because it does not only give us additional information about the conformation of linear chains near the surface, but also provides an entropic advantage for linear chains to be enhanced at the interface. In fact, the exposure of chain-ends of linear chains to empty space generates surface potentials of entropic origin [13,44]. The ratio of chain-ends of linear polymers $N_{ce}$ in a slab of thickness σ to the same number in a slab in the *bulk* $N_{ce}^{b}$, is written as

(8) $$P_{ce}=\frac{N_{ce}}{\left(N_{ce}^{b}\right)}$$

**Figure 6:**
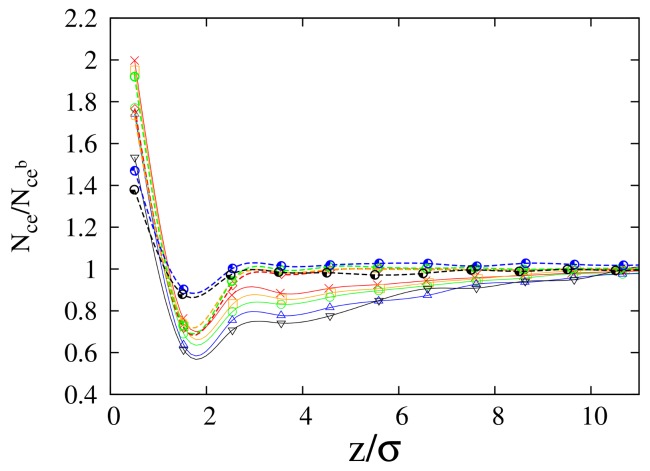
Normalized linear chain-ends for 100-mer (solid lines) and 10-mers (dashed lines) as a function of distance from the instantaneous interface, at concentrations $C_{0}$ = 10% (Red lines), 30% (Orange lines), 50% (Green lines), 70% (Blue lines) and 90% (Black lines).

Obviously, $P_{ce}$ signals chain-end enhancement when its value becomes greater than 1. Plots of $P_{ce}$ as a function of distance from instantaneous interface for both 100-mers and 10-mers are shown in Figure 6. Values of $P_{ce}$ for both chain lengths range from ≈1.4–2.0 at a distance z≈σ relative to the instantaneous interface. It is interesting to note that, very close to the interface, $P_{ce}$ for both chain lengths decreases as $C_{0}$ increases. Across all concentrations of the blend and irrespective of the chain length, the minimum of $P_{ce}$ appears located in the bin position z≈1.5σ. The position of the minimum is the same as the one reported in Figure 5. As a general trend, the bulk normalized number of chain-ends of the longer chain polymers (100-mers) is slightly higher than the one of short chains (10-mers) close to the interface. Regardless of the chain length, we generally observe that the number of chain ends at the surface decreases as we increase the cyclic concentration. Then, we can conclude that linear polymers will experience a decreasing entropy gain as the cyclic concentration is increased, regardless of the chain length. This evidence allows us to speculate that for short chains the entropy gain of linear chains, due to their chain ends, is a factor significantly affecting their enhancement at the interface. In fact, both the density profiles of Figure 1, and the information coming from the instantaneous interface of Figure 5 indicate a depletion of linear chains at the surface when the cyclic concentration is increased.

### 3.3. Surface Interaction Energy

**Figure 7:**
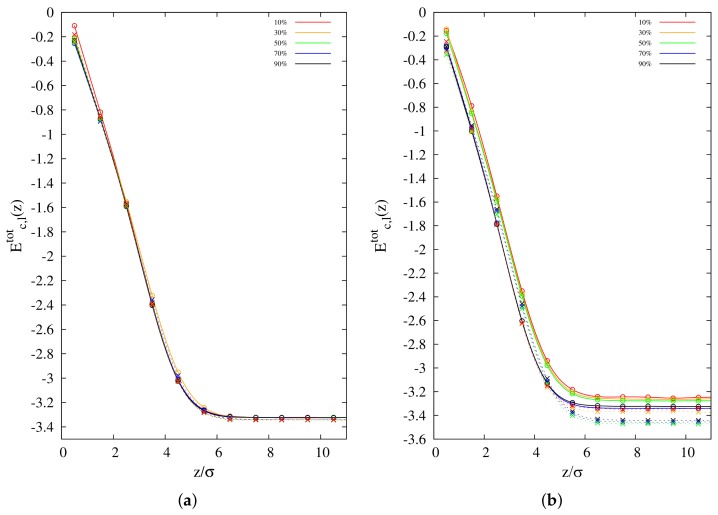
Interaction total energy per bead of cyclic and linear chains ($E_{c,l}$) as a function of distance from the interface at concentrations $C_{0}$ = 10%, 30%, 50%, 70% and 90% for: (**a**) 100-mers; and (**b**) 10-mers. Cyclic chains: solid lines; linear chains: dashed lines.

Plots of total interaction energies ($E_{l,c}^{tot}$) per bead of linear and cyclic chains as a function of distance from the vacuum/polymer interface are reported in Figure 7a,b. There is no noticeable dependence on concentration for the case of 100-mers, although cyclic chains display higher total interaction energies than linear ones at the interface, in agreement with what was observed before [14]. On the contrary, for the case of 10-mers, there is pronounced dependence on chain concentration. In general, the total energy per bead of cyclic chains at the interface decreases as cyclic concentration increases, while the total energy per bead of linear chains does not change significantly. $E_{l}^{tot}$ is clearly lower than $E_{c}^{tot}$ at the interface for lower cyclic concentrations, which confirms the picture of an enthalpic advantage for linear chains to be enhanced at the interface. Obviously, this enhanced enthalpic energy for linear polymers is associated with their peculiar topology: the presence of chain ends adds at least another channel of interaction, due to the presence of an additional site of covalent coordination. Moreover, the open configuration of chain ends is more likely to favor the increase in the number of contacts between polymer beads. However, the gap between the energies of the two polymer species shrinks as the cyclic concentration is increased, in agreement with the general observation confirmed by the previous quantities we calculated that the enhancement of linear chains at the interface decreases for higher cyclic concentrations.

### 3.4. Diffusion Coefficient

In this section, we analyze the diffusion coefficient of the polymer chains by first computing the mean square displacement (MSD) of the center-of-mass of polymer chains diffusing within slabs of thickness σ in the *z*-direction of the box, using Einstein’s relation [45]:

(9) $$\langle |\Delta \bar{R}{|}^{2}\rangle =2nD\Delta t$$

where *D* is the diffusion coefficient of a given polymer species, $\langle |\Delta \bar{R}|\rangle$ is the mean displacement vector of the center-of-mass of a molecule, Δt is the time over which the displacement occurs and *n* is the dimensionality of the space where molecules are moving through. Similar to the radius of gyration, the procedure described at the end of the subsection “Simulation details” is applied also for the evaluation of the MSD of the center of mass of polymers inside a slice of size σ along the *z*-direction. However, in this case the contribution to the diffusion coefficient arising from a generic polymer, whose center of mass lies within the slice, is considered only for the total time the polymer will stay inside the slice. Since the diffusion of polymer chains is expected to be different along the directions parallel and perpendicular to the interface, we hereby consider the parallel ($D^{∥}$) and perpendicular ($D^{⊥}$) components of *D*. These values are scaled with respect to their *bulk* values (i.e., $D^{∥}$/$D_{b}{}^{∥}$ and $D^{⊥}$/$D_{b}{}^{⊥}$) and denoted as $d^{∥}$ and $d^{⊥}$, respectively. The values of $D^{∥}$ and $D^{⊥}$ at the polymer/vacuum interface for both types of chains are shown in Table 3.

**Figure 8:**
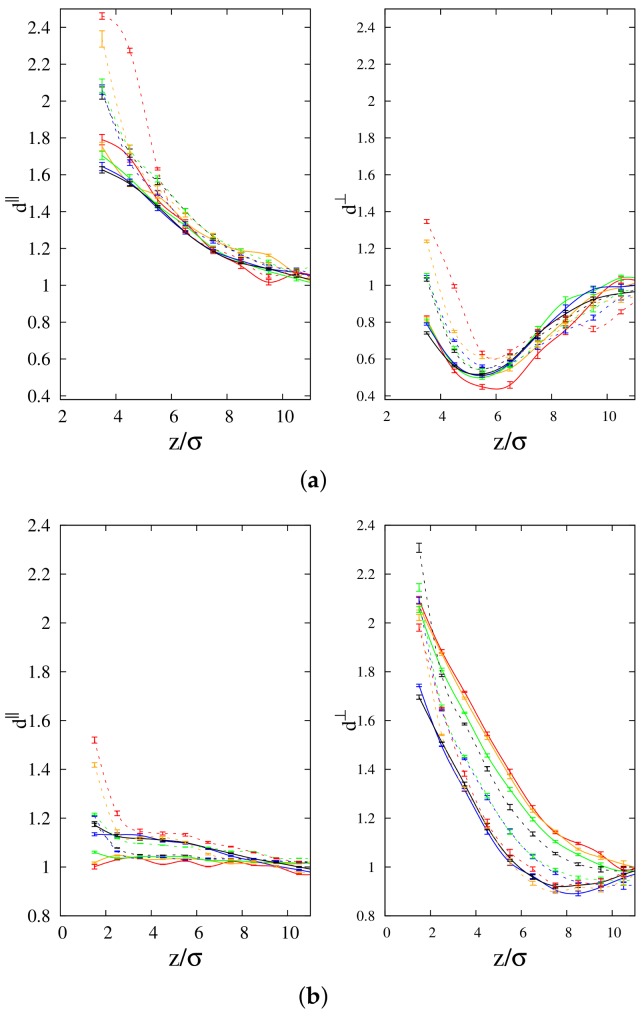
Variation of diffusion coefficient with distance from the interface for (**a**) 100-mers and (**b**) 10-mers of cyclic and linear chains at concentrations $C_{0}$ = 10% (Red lines), 30% (Orange lines), 50% (Green lines), 70% (Blue lines) and 90% (Black lines). Left and right panels represent parallel and perpendicular components of diffusion constant, respectively. Solid lines: cyclic chain profiles; dashed lines: linear chain profiles.

In general, for long chains (100-mers) the mobility along the parallel direction to the interface of both the linear and cyclic chains (see left panel of Figure 8a) is higher than in the *bulk* for all concentrations, and for both chain lengths. In particular, the disparity between *bulk* and surface diffusion coefficients is quite high for the parallel component ($d^{∥}$) of both polymer species. For 100-mers, the parallel diffusion coefficient of linear polymers is observed to be systematically higher than that of cyclic chains, and for both polymer species it decreases as a function of the cyclic concentration. We also note that the gap between the diffusion coefficients of the two polymer species at the interface tends to decrease when the cyclic concentration is increased. If we look at the mobility along the parallel direction to the interface for short chains (10-mers) (see left panel of Figure 8b), we observe a significant enhancement of the diffusion coefficient only for the smaller cyclic concentration. Interestingly, while the diffusion coefficient of linear polymers decreases with increasing cyclic concentration, the opposite trend is observed for cyclic polymers. This feature makes the gap at the interface between the diffusion coefficient of the two polymer species to shrink almost to zero for the higher cyclic concentration, while the diffusion coefficient of linear polymers appears also in this case to be systematically higher than the one of cyclic polymers.

The perpendicular component ($d^{⊥}$) of the diffusion coefficient for long chains (100-mers, see right panel of Figure 8a) partly shows the same qualitative behavior of the corresponding parallel component (see left panel of Figure 8a), i.e., the diffusion coefficient of linear polymers is observed to be systematically higher than that of cyclic chains, and for both polymer species it decreases as a function of the cyclic concentration. However, the values of $d^{⊥}$ at the interface are not significantly larger than the *bulk* value for the linear polymers, while they are even smaller than in the case of cyclic polymers. In fact, an interesting feature develops for both polymer species, that is $d^{⊥}$ has a non-monotonic behavior as a function of the distance from the interface, and a minimum is observed that shifts from approximately 6σ for low cyclic concentrations to nearly 5σ for high cyclic concentrations. The location of the minimum is the same for the two polymer species at the same concentration, and it causes the cyclic chains to experience an even slower diffusion as compared to the *bulk* (the minimum falls at values significantly below 1). In the case of short chains (10-mers, see right panel of Figure 8b), the perpendicular component ($d^{⊥}$) of the diffusion coefficient shows the opposite trend as compared to the corresponding parallel component (see left panel of the same figure). Nonetheless, $d^{⊥}$ for linear chains increases when we consider higher cyclic concentrations, while for cyclic chains it does decrease at higher cyclic concentration. In this case, we observe ,similarly as for the parallel component of the diffusion coefficient of long chains (see right panel of Figure 8a), quite higher mobility at the interface as compared to the *bulk*. However, there is a clear difference between long and short chains in the qualitative behavior of $d^{⊥}$ as a function of cyclic concentration, because in the case of $d^{∥}$ of 100-mers the mobility gap between the two polymer species seems to slightly decrease upon increasing the cyclic concentration. On the other hand, the gap of $d^{⊥}$ for the two polymer species in the case of 10-mers becomes larger as the cyclic concentration is increased because of the trend of $d^{⊥}$ we noted earlier in the right panel of Figure 8b. We also observe an important feature which relates to the blend ratio $D_{l}^{∥,⊥}/D_{c}^{∥,⊥}$ between diffusion coefficients of linear and cyclic chains. At any given concentration near the interface of the blend, this ratio is not less than 1.00 but does not exceed 1.38, which agrees with the findings of Alatas et al. [46] that $D_{l}/D_{c}$ must not exceed a value of 1.40. Obviously, on average, linear chains exhibit self-diffusion coefficients that are higher than those of cyclic chains. The slower diffusion of cyclic chains can be attributed to topological restrictions acting on the conformation of the cyclic molecules [47].

## 4. Conclusions

The surface properties of polymer blends containing linear and cyclic chains at different cyclic concentrations were investigated. The effect of molecular weight of the polymers on their surface properties was also investigated through simulation of short (10-mers), and relatively long (100-mers) long chains.

For long polymer chains, we found that cyclic chains are more enhanced at the interface, but the concentration of cyclic chains becomes less enhanced upon increase of their concentration. The parallel to the interface component of the radius of gyration of both the polymer species increased with cyclic concentration, and this effect was more significant for cyclic chains. The same behavior is observed for the perpendicular component to the interface of the radius of gyration. In general, the results reported for the two components of the radius of gyration indicate that long cyclic chains exhibit a more evident tendency to be elongated parallel to the interface, as the concentration of cyclic chains increases. The total interaction energy per bead did not show a noticeable dependence on concentration, but even the tiny differences observed between the energy of cyclic and linear chains at the interface, suggest that linear chains have lower energy than cyclic chains, which is a consequence of the higher flexibility of the linear polymers. Both the parallel and perpendicular components of the diffusion coefficient of linear chains at the interface are observed to be systematically higher than the ones of cyclic chains. In general, the diffusion of both polymer species decreases with increasing cyclic concentration.

In the case of short polymer chains, we found that the concentration of linear chains was more enhanced at the interface at low cyclic concentrations than at high concentrations, and at high cyclic concentrations no appreciable preferential absorption of linear chains was observed at the interface. This evidence provides a computational support of the reason why enhancement of linear polymers at the interface was observed for lower cyclic concentrations only. The parallel component of the radius of gyration of both the polymer species increased as a function of cyclic concentration, similar to the long polymer chain case. However, our results indicate that linear chains lose more conformational entropy than cyclic chains when the cyclic concentration increases (see left panel of Figure 3, where the gap between the parallel components of the radius of gyration of the two polymer species is reported), thus providing a microscopic understanding of the corresponding trend of enhancement of the density of cyclic chains at the interface. Near the interface, the range of variation of the transverse and parallel components of the radius of gyration was found to be similar for both the polymer species. The total energy of interaction per bead of cyclic chains shows a pronounced dependence on chain concentration for short chains. Finally, we observed a significant enhancement of the mobility along the parallel direction to the interface only for the smaller cyclic concentration. Furthermore, the mobility of linear polymers decreased upon increase of cyclic concentration, while the mobility of cyclic polymers increased. On the other hand, the perpendicular component of the diffusion coefficient shows the opposite trend upon increase of cyclic concentration.

This study allowed us to get a better understanding of how the enhancement of either of the two polymer species changes as a function of the concentration for polymer blends of two topologically-different species, in the presence of a low-energy interface (vacuum).

## Figures and Tables

**Table 1:** Values of *bulk* density of linear and cyclic chains for 10-mers and 100-mers. Errors are on the last digit.

% Cyclic concentration	10-mers		100-mers	
	$\rho_{\mathrm{Linear}}^{0}$	$\rho_{\mathrm{Cyclic}}^{0}$	$\rho_{\mathrm{Linear}}^{0}$	$\rho_{\mathrm{Cyclic}}^{0}$
10	3.13	0.35	3.23	0.33
30	2.44	1.06	2.54	1.02
50	1.74	1.78	1.87	1.69
70	1.05	2.48	1.15	2.41
90	0.35	3.20	0.40	3.17

**Table 2:** Average *bulk* radii of gyration for linear and cyclic chains. Errors are on the last digit.

% Cyclic concentration	100-mers						10-mers					
	Linear chain			Cyclic chain			Linear chain			Cyclic chain		
	$R_{g,b}$	$R_{g,b}^{\left|\right|}$	$R_{g,b}^{⊥}$	$R_{g,b}$	$R_{g,b}^{\left|\right|}$	$R_{g,b}^{⊥}$	$R_{g,b}$	$R_{g,b}^{\left|\right|}$	$R_{g,b}^{⊥}$	$R_{g,b}$	$R_{g,b}^{\left|\right|}$	$R_{g,b}^{⊥}$
10	5.06	2.84	2.79	3.77	2.18	2.08	1.45	0.81	0.79	1.14	0.66	0.64
30	5.10	2.86	2.82	3.68	2.11	2.07	1.44	0.81	0.79	1.14	0.65	0.64
50	5.09	2.87	2.78	3.63	2.08	2.04	1.44	0.81	0.79	1.14	0.65	0.64
70	5.01	2.82	2.76	3.57	2.05	2.00	1.26	0.71	0.70	1.22	0.70	0.68
90	5.04	2.84	2.81	3.51	2.01	1.98	1.20	0.68	0.67	1.17	0.67	0.65

**Table 3:** Parallel and perpendicular components of surface diffusion constant for 100-mers and 10-mers chains computed from the slope of MSD using Einstein’s relation. Errors are on the last digit.

% Cyclic concentration	100-mers				10-mers			
	Linear chain		Cyclic chain		Linear chain		Cyclic chain	
	$D_{\mathrm{surface}}^{\left|\right|}$	$D_{\mathrm{surface}}^{⊥}$	$D_{\mathrm{surface}}^{\left|\right|}$	$D_{\mathrm{surface}}^{⊥}$	$D_{\mathrm{surface}}^{\left|\right|}$	$D_{\mathrm{surface}}^{⊥}$	$D_{\mathrm{surface}}^{\left|\right|}$	$D_{\mathrm{surface}}^{⊥}$
10	0.10	0.11	0.05	0.05	6.00	1.19	3.11	0.63
30	0.11	0.12	0.06	0.06	5.89	1.26	3.10	0.64
50	0.12	0.12	0.06	0.06	5.80	1.27	3.09	0.70
70	0.12	0.12	0.07	0.07	2.25	1.28	1.09	0.80
90	0.15	0.15	0.08	0.08	2.18	1.28	1.07	1.00
